# Histological analysis of the medial gastrocnemius muscle in young healthy children

**DOI:** 10.3389/fphys.2024.1336283

**Published:** 2024-04-08

**Authors:** Anke Andries, Jorieke Deschrevel, Karen Maes, Nathalie De Beukelaer, Marlies Corvelyn, Lauraine Staut, Hannah De Houwer, Domiziana Costamagna, Stefaan Nijs, Willem-Jan Metsemakers, Elga Nijs, Greet Hens, Eva De Wachter, Sandra Prinsen, Kaat Desloovere, Anja Van Campenhout, Ghislaine Gayan-Ramirez

**Affiliations:** ^1^ Laboratory of Respiratory Diseases and Thoracic Surgery, Department of Chronic Diseases and Metabolism, KU-Leuven, Leuven, Belgium; ^2^ Neurorehabilitation Group, Department of Rehabilitation Sciences, KU-Leuven, Leuven, Belgium; ^3^ Stem Cell and Developmental Biology, Department of Development and Regeneration, KU-Leuven, Leuven, Belgium; ^4^ Pediatric Orthopedics, Department of Development and Regeneration, KU-Leuven, Leuven, Belgium; ^5^ Exercise Physiology Research Group, Department of Movement Sciences, KU-Leuven, Leuven, Belgium; ^6^ Department of Trauma Surgery, University Hospitals Leuven, Leuven, Belgium; ^7^ Department of Ear Nose Throat, University Hospitals Leuven, Leuven, Belgium; ^8^ Department of Orthopaedic Surgery, University Hospitals Leuven, Leuven, Belgium

**Keywords:** boys, girls, muscle capillaries, muscle fiber size and proportion, myosin heavy chain, satellite cells, fiber size variability

## Abstract

**Introduction:**

Histological data on muscle fiber size and proportion in (very) young typically developing (TD) children is not well documented and data on capillarization and satellite cell content are also lacking.

**Aims:**

This study investigated the microscopic properties of the medial gastrocnemius muscle in growing TD children, grouped according to age and gender to provide normal reference values in healthy children.

**Methods:**

Microbiopsies of the medial gastrocnemius (MG) muscle were collected in 46 TD boys and girls aged 2–10 years subdivided into 4 age groups (2–4, 4–6, 6–8 and 8–10 years). Sections were immunostained to assess fiber type cross-sectional area (fCSA) and proportion, the number of satellite cells (SC), capillary to fiber ratio (C/F), capillary density for type I and II fiber (CFD), capillary domain, capillary-to-fiber perimeter exchange index (CFPE) and heterogeneity index. fCSA was normalized to fibula length^2^ and the coefficient of variation (CV) was calculated to reflect fCSA intrasubject variability.

**Results:**

Absolute fCSA of all fibers increased with age (r = 0.72, *p* < 0.001) but more in boys (+112%, *p* < 0.05) than in girls (+48%, *p* > 0.05) Normalized fCSA, CV and fiber proportion did not differ between age groups and gender. C/F was strongly correlated with age in boys (r = 0.83, *p* < 0.001), and to a lesser extent in girls (r = 0.37, *p* = 0.115), while other capillary parameters as well as the number of SC remained stable with increasing age in boys and girls.

**Discussion:**

This study provides reference values of histological measures in MG according to age in normally growing boys and girls. These data may be used as a reference to determine disease impact and efficacy of therapeutic approach on the muscle.

## 1 Introduction

Skeletal muscles are highly plastic and can adapt their mass and fiber size in response to physiological and pathological conditions. Stimuli such as changes in neuromuscular stimulation, mechanical loading, and exercise as well as hormone levels have an effect on the architecture of skeletal muscle (Qaisar et al., 2016). An increase in muscle mass and fiber size characterizes muscle growth, also called hypertrophy. This occurs on several occasions as a natural phenomenon during the development from birth to adult, but also as an adaptation in response to overload, for example, after strength training or anabolic stimulation (Lipina and Hundal, 2017). In the same line, atrophy which is a decrease in muscle mass and fiber size is observed during aging, starvation, catabolic stimulation, bed rest and pathological stages like diabetes, cancer, and a loss of neural input (Lipina and Hundal, 2017). Importantly, muscle fiber cross-sectional area is linked to the maximum force-generating capacity of the skeletal muscle and the fiber type proportion is related with endurance and fatigue resistance. The composition of a given skeletal muscle is related to its function and is depending on its fiber type proportion with the type I fibers generating the lowest force and being fatigue resistant while the type IIx fibers produce the largest force but are fast fatigable.

Under normal conditions, satellite cells which represent the stem cells of skeletal muscles, reside in a quiescent state between the sarcolemma and the basal lamina. These cells are essential for muscle growth, especially for the post-natal development (Forcina et al., 2019). In particular, muscle growth during childhood development implies a continuous activation of the satellite cells as well as their proliferation and differentiation into new myofibers (Pallafacchina et al., 2013; Carlson, 2022). Muscle growth results from an increase in fiber cross-sectional area and length. There are currently no data on absolute numbers of satellite cells throughout childhood notably at young age or according to gender. Similarly, data on fiber cross-sectional areas in growing muscle are poorly documented.

Moreover, skeletal muscles are highly vascularized and capillaries are essential for the transport of nutrients, hormones and oxygen to the muscle and the extraction of heat, metabolites and waste (Barnouin et al., 2017). The number of capillaries is determined by the muscle fiber size and muscle fiber type and not by the muscle oxidative capacity (Wüst et al., 2009). As such, muscle growth during childhood development where fiber size increases with increasing age, is associated with concomitant increase in capillary network in order to maintain appropriate tissue supply. However, data on capillarization in skeletal muscle throughout childhood have been poorly investigated.

Muscle biopsy analysis is often used for the diagnosis of neuromuscular disorders, the follow-up of disease progression and the assessment of the response on therapeutic interventions. For that purpose, it is imperative to have knowledge on muscle features (e.g., fiber size and proportion, number of capillaries and satellite cells) in healthy individuals grouped according to age and gender. Over the last decades, extensive research has been performed in healthy adults and has shown that for a given muscle, fiber size and proportion differ between male and female as well as between upper and lower limb muscles (Jennekens et al., 1971; Simoneau and Bouchard, 1989; Staron et al., 2000). By contrast, very little is known on the muscles of healthy children (Sallum et al., 2013), although this information is highly relevant keeping in mind that the muscle fiber size increases with growth during childhood. This implies that the normal values of (very) young children differ from normal values of older children and using inappropriate age range data for comparison may lead to data misinterpretation. Additional drawbacks of the performed studies in children, for example, are 1) the age range of included subjects with scarce data on very young children (Valentine et al., 2019; Esbjörnsson et al., 2022; Hester et al., 2022), 2) the use of data from autopsy specimen (Jennekens, Tomlinson, and Walton, 1971; Johnson et al., 1973; Lexell et al., 1992), 3) from very active children (Esbjörnsson et al., 2021), 4) or from subjects who underwent surgery that limited their physical activity (Domenighetti et al., 2018), 5) the absence of data on fiber size for the leg muscle (Kriketos et al., 1997), 6) the lack of information on effect of gender if any (Lee et al., 2006; Delhaas et al., 2013) etc., … It seems that sex related differences in fiber pattern development occur during the transition from childhood to adulthood (Esbjörnsson et al., 2021), but whether sex related differences are already present at younger age is not yet known. Furthermore, while only a couple of studies have been performed on the satellite cells in healthy adults (Kadi et al., 1999; Carlson et al., 2009; Mackey et al., 2009; Mackey et al., 2010; Bankolé et al., 2013), there are almost no data on healthy young children (Saito, 1985; Maier and Bornemann, 1999; Verdijk et al., 2014; Corvelyn et al., 2020). Moreover, similarly to fiber size, data on lower leg muscles in very young children are scarce (Saito, 1985; Sallum et al., 2013). Finally, capillarization in healthy children has been poorly explored and only refers to data obtained from *postmortem* samples (Hepple RT, 1997; Pontén and Stål, 2007).

Up to now, comprehensively investigations of muscle characteristics (fiber cross-sectional area, fiber type proportion, number of satellite cells, capillarization) in healthy young children grouped according to age and gender are missing, although this knowledge seems warranted in order to avoid any misinterpretation due to using inappropriate control data. In this study, we aimed at investigating the microscopic muscle characteristics of the medial gastrocnemius muscle of typically developing boys and girls aged 2–10 years. Micro biopsies of the medial gastrocnemius were collected under general anesthesia and histological staining’s were performed to address: 1) fiber cross-sectional area and fiber type proportion, 2) capillarization, 3) number of satellite cells.

## 2 Materials and methods

### 2.1 Participants and ethics

Typically developing children (TD) with an age range from 2 till 10 years planned for elective post trauma surgery (removal of osteosynthesis material after upper limb trauma) or ear-nose-throat surgery were recruited from Traumatology Unit or the Ear-Nose-Throat Unit of the University Hospital of Leuven (Leuven, Belgium). The exclusion criteria included history of lower limb pathology or surgery, a history of neurological or musculoskeletal disorders, or the participation in sports activities for more than 3 h/week. Children were subsequently subdivided into different age groups: 2–4 years, 4–6 years, 6–8 years and 8–10 years. The study protocol was approved by the Ethical Committee of the University Hospital Leuven/KU Leuven, Belgium (S62645). Parents or legal guardians gave written consent for the participation of the child in the study prior to the biopsy collection.

### 2.2 Biopsy collection and storage

The muscle micro biopsies were collected when the children underwent orthopedic, trauma or Ear-Nose-Throat surgery that required general anesthesia. Percutaneous muscle micro biopsies from the muscle belly of the medial gastrocnemius (MG) were collected under ultrasound guidance using a 16-gauge micro biopsy needle (Bard^®^ Mission™ Disposable Core Biopsy Instrument 16G x 10 cm–Semi-Automatic). For that purpose, the leg was positioned with a slightly abducted and flexed hip (knee in 60°–90° flexion and ankle in resting position resulting in average 10° of plantar flexion position). The micro biopsy samples were snap-frozen in liquid nitrogen-cooled isopentane and stored at −80°C till further use.

### 2.3 Histological and immunohistochemical analysis

The biopsies were cut in 5 μm thick cryosections with a cryostat at −20°C (CryostarTM NX 70 Thermo Fisher Scientific), and then transferred on a charged slide (Superfrost plus, VWR) and stored at −20°C.

#### 2.3.1 Muscle fiber cross-sectional area and fiber type proportion

The slides were incubated for 1 h at room temperature with a 10% goat serum blocking solution (Thermo Fisher Scientific) followed by an overnight incubation at 4°C with a primary antibody cocktail against laminin (Abcam, ab11575), myosin heavy chain (MHC)-I (BA-F8,DSHB), MHC-IIa (SC-71, DSHB), MHC-IIx (6H1, DSHB) (adapted from Bloemberg and Quadrilatero (2012)). Hereafter, the slides were washed 3 × 5 min with 1xPBS (7011044, Thermo Fisher Scientific) and incubated with a secondary antibody cocktail (Alexa Fluor 680, 350, 488, and 555, Thermo Fisher Scientific) for 1 h at room temperature. The slides were washed 3 × 5 min in 1xPBS and mounted using ProLong^®^ Gold antifade reagent (Molecular Probes, Thermofisher). Pictures were taken with a fluorescence microscope (20x objective, Leica DMi8). The fiber cross-sectional area (fCSA), minimal Feret diameter (MiniFeret) and proportion of the type I (%MHC-I), type IIa (%MHC-IIA) and type IIx (%MHC-IIX) fibers were determined using ImageJ. The distribution frequency of fCSA, binned in 500 μm^2^ intervals, was calculated and visualized as histograms.

The coefficient of variation (CV) was calculated using:

$$CV\%=\left(\frac{SD}{Mean}\right)*100$$

and the relative contribution of each fiber type to the total surface were calculated using:

$$\frac{\left(\text{CSA}\;\text{MHC}‐I,\text{IIa}\;\text{or}\;I\text{Ix}\;*\;\%M\text{HC}‐I,\text{IIa}\;\text{or}\;\text{II}x\right)}{\left(\left(\text{CSA}\;\text{MH}C‐I\;*\;\%\text{MH}C‐I\right)+\left(\text{CSA}\;\text{MH}C‐I\text{Ia}\;*\;\%M\text{HC}‐\text{II}a\right)+\left(C\text{SA}\;\text{MHC}‐I\text{Ix}\;*\;\%\text{MHC}‐\text{IIx}\right)\right)}$$

A normalization technique was applied to eliminate the effect of growth on the fCSA. We selected the best normalization factor based on the smallest slope of the relation (simple linear regression line) between the normalized fCSA and age, to entirely exclude the impact of anthropometric growth. The normalization factor Fibula^2^ was found to be the most efficient and was therefore selected to normalize fCSA. For that purpose, the fCSA was divided by the squared fibula length to obtain normalized fCSA values.

#### 2.3.2 Capillarization

The slides were fixed in cold acetone for 10 min at 4°C. After fixation, the slides were washed 3 × 5 min in 1xPBS (7011044, Thermo Fisher Scientific) and blocked for 1 h at room temperature with a 10% goat serum (Thermo Fisher Scientific) blocking solution. The slides were incubated overnight at 4°C with a primary antibody cocktail directed to MHC type I (BA-F8, DSHB) and capillaries (CD-31, Abcam ab28364) whereafter they were washed 3 × 5 min in 1xPBS (Thermo Fisher Scientific). The slides were incubated for 1 h at room temperature with a secondary antibody cocktail (Alexa Fluor 488 and 555, Thermo Fisher Scientific) and then washed 3 × 5 min in 1xPBS. After washing, the slides were incubated overnight at 4°C with a primary antibody against laminin (Abcam, ab11575) and washed 3 × 5 min in 1xPBS. The slides were then incubated for 1 h at room temperature with a secondary antibody Alexa 680 (Thermo Fisher Scientific). After washing 3 × 5 min in 1xPBS, the slides were incubated for 1 min with DAPI (D1306, Thermo Fisher Scientific). After the final washing step of 3 × 5 min in 1xPBS, the slides were mounted with Prolong Gold antifade reagent (Molecular Probes, Thermo Fisher Scientific). The slides were observed, and pictures were taken with a fluorescence microscope (40x objective, Leica DMi8).

The samples were quantified with the applications Btablet and Anatis (BaLoH Software, NL). The following parameters were determined: 1) the capillary fiber density (CFD) calculated as the capillaries per mm^2^ muscle tissue for type I and type II fibers, 2) the capillary to fiber ratio (C/F), 3) the capillary domain which is the region with an equal distance between two nearby capillaries, 5) the heterogeneity index (LogSD) for the distribution of the capillaries, 6) the Capillary-to fiber Perimeter Exchange (CFPE) index by dividing the CF of an individual fiber with its perimeter (Hepple RT, 1997; Pontén and Stål, 2007; Ballak et al., 2016).

#### 2.3.3 Number of satellite cells

The slides were fixed in cold acetone for 10 min at 4°C. After fixation, they were washed 3 × 5 min in 1xPBS (Thermo Fisher Scientific) and blocked for 1 h at room temperature with a 10% goat serum (Thermo Fisher Scientific) blocking solution. The slides were incubated overnight at 4°C with a primary antibody cocktail against laminin (Abcam, ab11575), MHC-I (BA-F8, DSHB) and Pax7 (DSHB), thereafter they were washed 3 × 5 min in 1xPBS (7011044, Thermo Fisher Scientific). They were then incubated for 1 h at room temperature with a secondary antibody cocktail (Alexa Fluor 680,350 and 488, Thermo Fisher Scientific) and then washed 3 × 5 min in 1x PBS. Following washing, the slides were incubated for 1 min at room temperature with DAPI (D1306, Thermo Fisher Scientific), and washed 3 × 5 min with 1xPBS. Finally, the slides were mounted with Prolong Gold antifade reagent (Molecular Probes, Thermo Fisher Scientific). The slides were observed, and pictures were taken with a fluorescence microscope (40x objective, Leica Dmi8). The samples were quantified with ImageJ by counting the number of SC, MHC type I fibers and MHC type II fibers. The identification of the SC was done by the co-localization of Pax7 and DAPI. ImageJ provided information about the number of satellite cells/100 fibers for MHC type I and MHC type II.

### 2.4 Statistical analysis

Normality was tested by a Shapiro wilk test. For normally distributed data an one-way ANVOVA was used, followed by a multiple comparison test to compare different age groups. For not normally distributed data, a Kruskal Wallis test was used, followed by a pairwise comparison using Dunn’s (1964) to compare different age groups. To assess the differences between boys and girls in a certain age group, a Mann-Whitney test was performed. When the data was normally distributed a Pearson correlation test was used to address correlations between the different age groups. While a Spearman test for was used to address correlation of not normally distributed data between the different parameters. Frequency distribution differences between groups were tested with a chi-square test. Moreover, for all data a Bonferroni correction was applied to correct for multiple testing. The data are presented as mean and standard deviation for normally distributed data and as median and interquartile range for not normally distributed data. Statistical analysis was performed with the statistical software package SPSS software platform (Version 28.0.1.1, IBM, Armonk, NY, United States). Graphs were created with Graphpad prism (software version 9.2.0, San Diego, CA, United States).

## 3 Results

### 3.1 Population characteristics

The study sample included 46 TD children aged 6.5 ± 2 years (range 2.1–9.8 years, 22 girls and 24 boys), with a body weight and body height of 22.0 ± 5.6 kg and 120 ± 15 cm and a tibia and fibula length of 27.9 ± 4.6 cm and 25.7 ± 4.3 cm, respectively. There were no differences between boys and girls concerning age (6.3 ± 2.1 and 6.6 ± 1.8 years, respectively), body height (119 ± 16 and 121 ± 13 cm, respectively), body weight (21.6 ± 6.1 and 22.1 ± 5.2 kg, respectively), tibia length (27.6 ± 5 and 28.3 ± 4.5 cm, respectively) and -fibula length (25.6 ± 4.6 and 25.8 ± 4.2 cm, respectively). The participant characteristics are summarized in Table 1.

**TABLE 1:** Population characteristics of boys (upper) and girls (lower) according to age groups. Individual data as well as means and standard deviation are depicted.

Age group (yr)	Boys	Age (years)	Ethnicity	Body weight (kg)	Body length (cm)	Tibia length (cm)	Fibula length (cm)
2–4	1	2.10	Caucasian	12.0	83	18.0	17.0
	2	2.83	Caucasian	14.0	92	20.5	19.0
	3	3.35	Latin American	15.0	100	21.5	19.5
	4	3.49	Caucasian	16.5	103	23.0	20.8
	5	3.87	Caucasian	14.0	103	23.5	22.0
4–6	6	4.64	Caucasian	16.3	106	23.5	21.0
	7	4.93	Caucasian	17.8	110	25.0	24.0
	8	5.21	Asian	17.3	113	23.5	22.0
	9	5.61	Caucasian	20.7	115	28.5	26.0
	10	5.67	African	21.0	115	27.5	25.5
6–8	11	6.10	Caucasian	22.0	127	26.0	24.5
	12	6.43	Caucasian	18.0	125	NA	NA
	13	6.66	North African	25.0	127	28.6	27.0
	14	6.77	Caucasian	25.0	125	30.0	27.0
	15	7.06	Caucasian	23.9	127	28.0	26.0
	16	7.29	Caucasian	25.0	122	30.0	26.5
	17	7.77	Caucasian	21.0	125	28.5	26.0
8–10	18	8.00	Caucasian	23.8	128	29.0	27.5
	19	8.63	Caucasian	23.0	145	29.5	27.5
	20	8.70	Caucasian	32.0	140	36.0	33.0
	21	8.87	Caucasian	31.1	140	32.0	29.7
	22	9.10	Caucasian	29.0	140	35.1	33.0
	23	9.55	Caucasian	33.0	130	34.0	32.2
	24	9.80	Caucasian	30.0	138	34.0	32.5
	Mean ± SD	6.31 ± 2.10	—	21.6 ± 6.1	119 ± 16	27.6 ± 4.8	25.6 ± 4.6

Age group (yr)	Girls	Age (years)	Ethnicity	Body weight (kg)	Body length (cm)	Tibia length (cm)	Fibula length (cm)
2–4	1	3.34	Caucasian	14.0	98	21.0	18.5
	2	3.52	Caucasian	15.0	102	22.4	20.8
4–6	3	4.16	Caucasian	14.0	104	25.6	23.2
	4	4.51	Caucasian	16.0	104	21.0	19.5
	5	4.77	Caucasian	19.5	107	24.8	23.0
	6	5.04	Caucasian	18.0	108	25.0	23.0
	7	5.58	Caucasian	20.0	120	25.5	22.0
	8	5.94	Caucasian	18.0	116	26.0	24.0
6–8	9	6.25	Caucasian	20.0	117	26.0	24.5
	10	6.43	Caucasian	24.0	122	28.0	25.0
	11	7.01	Caucasian	24.9	124	29.0	26.0
	12	7.10	Caucasian	27.0	127	NA	NA
	13	7.12	Caucasian	20.3	126	29.0	26.0
	14	7.36	Caucasian	22.4	125	29.0	27.0
	15	7.47	Caucasian	23.5	134	31.0	28.5
	16	7.51	Caucasian	24.0	123	27.5	25.0
	17	7.73	Caucasian	30.0	135	33.0	30.5
8–10	18	8.02	Caucasian	25.0	135	33.2	31.0
	19	8.23	Caucasian	25.5	NA	35.0	32.5
	20	9.30	Caucasian	23.4	132	31.0	28.0
	21	9.39	Caucasian	30.5	138	34.5	30.5
	22	9.43	Caucasian	32.0	145	36.0	33.5
	Mean ± SD	6.60 ± 1.82	—	22.1 ± 5.2	121 ± 13	28.3 ± 4.5	25.8 ± 4.2

NA, not applicable.

The subdivision into age group resulted in a group of 7 participants for the age group 2–4 years (5 boys, 2 girls), 11 participants for the age group 4–6 years (5 boys, 6 girls), 16 participants for the age group 6–8 years (7 boys, 9 girls) and 12 participants for the age group 8–10 years (7 boys, 5 girls). There were no differences in age, body height, body weight, tibia and fibula length between boys and girls within each age group.

**FIGURE 1:**
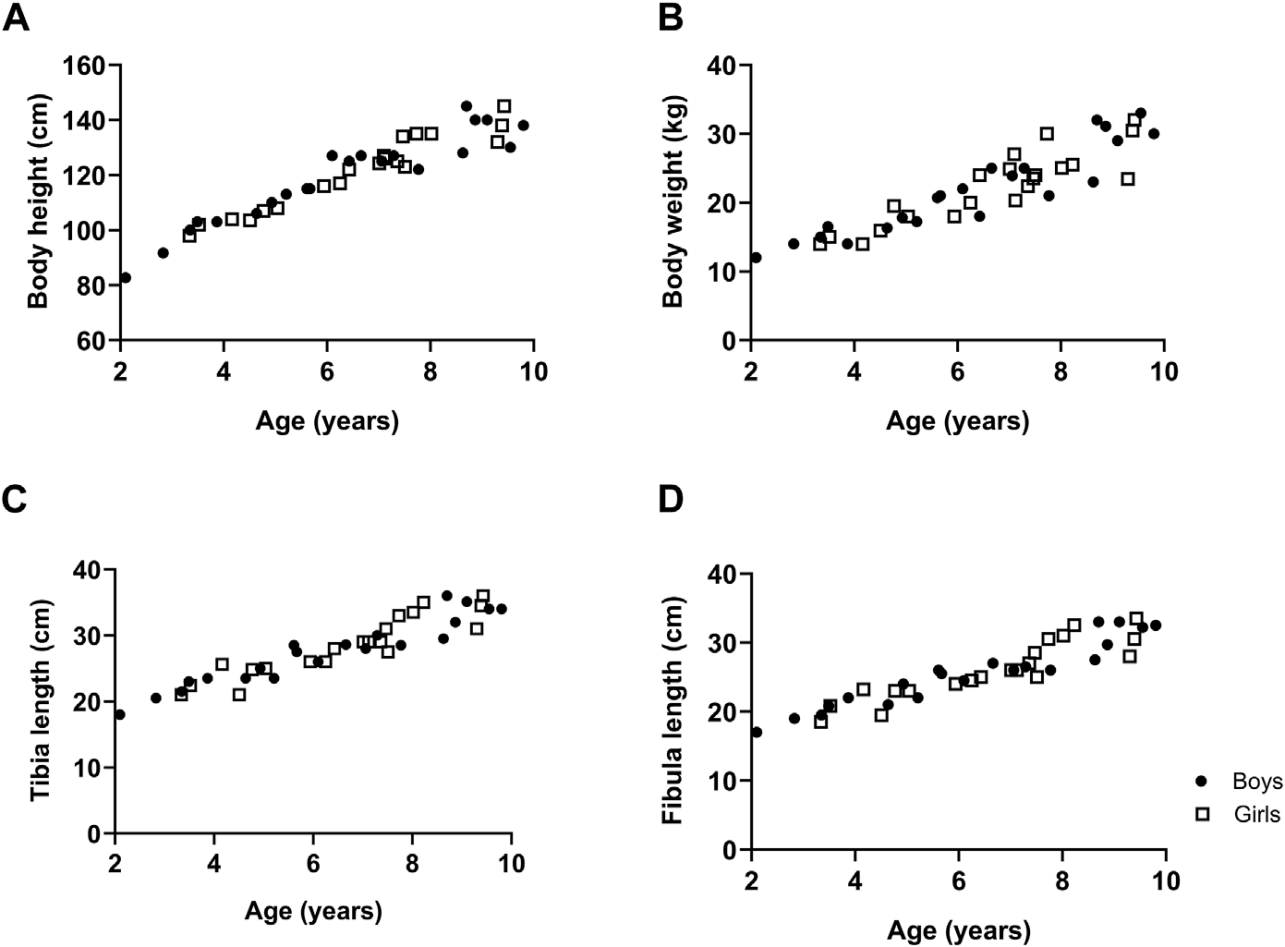
The growth of typically developing children with respect to age. Body height **(A)**, body weight **(B)**, tibia length **(C)** and fibula length **(D)**. *n* (2–4 years) = 7; *n* (4–6 years) = 11; *n* (6–8 years) = 16; *n* (8–10 years) = 12. Symbols: circles = boys; squares = girls. Each symbol represents individual value.

The body height, body weight, tibia length and fibula length with respect to age are represented in Figure 1 for boys and girls. Data showed that body height and body weight progressively increased with age to the same extent in both boys and girls as did tibia and fibula length (Figure 1).

### 3.2 Muscle fiber type cross-sectional area and fiber type proportion

**FIGURE 2:**
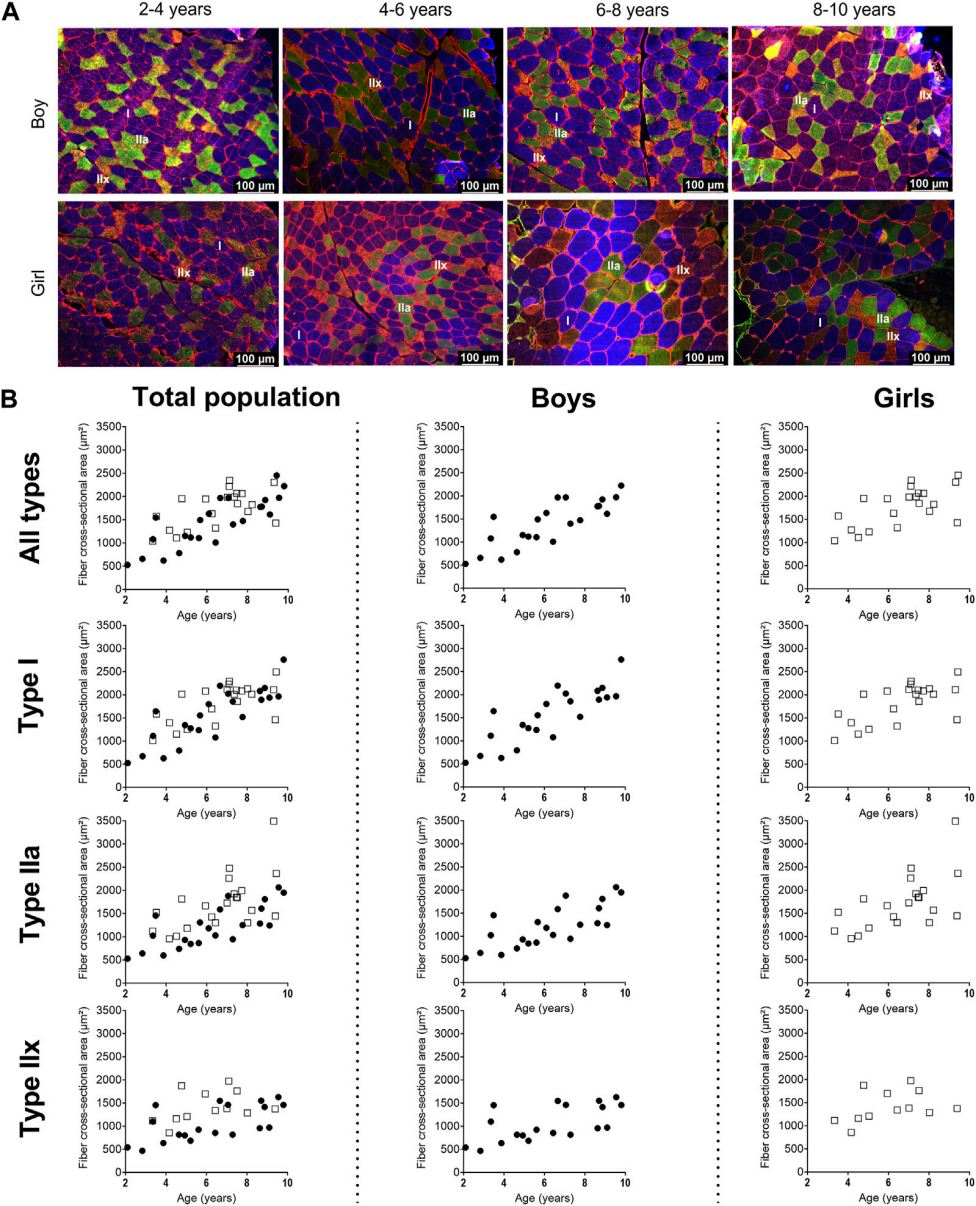
**(A)** Representative examples of fiber type staining in the medial gastrocnemius muscle of boys (upper panels) and girls (lower panels) according to age groups. Children aged 3 years, 5 years, 7 years and 8 years. Type I fibers are in blue (I), type IIa fibers are in green (IIa), type IIx fibers are in orange (IIx), and laminin surrounds the cells in red. Scale represents 100 µm. **(B)** Correlation between absolute fiber cross-sectional area of the medial gastrocnemius muscle in the total population (left panels), the boys (middle panels), and the girls (right panels) for all fiber types, the type I fibers, the type IIa and the type IIx fibers according to age. *n* (2–4 years) = 7; *n* (4–6 years) = 10; *n* (6–8 years) = 15; *n* (8–10 years) = 11. symbols: circles = boys; squares = girls. Each symbol represents individual value.

Representative examples of MHC staining showing the different fiber types in the MG are depicted in Figure 2A for boys and girls according to age range. For the assessment of the fiber type CSA and proportion, we counted on average 325 ± 200 fibers (for boys: 371 ± 233 fibers and for girls: 277 ± 149 fibers). The number of counted fibers ranged from 148–1,022 for boys, and from 147–769 for girls.

#### 3.2.1 Fiber type cross sectional area and coefficient of variation

For the total population, the absolute fCSA of all fibers significantly increased with increasing age (r = 0.72, *p* < 0.001), with larger fibers in the 6–8 age group and 8–10 age group compared to the 2–4 age groups (+86%, *p* < 0.005) and larger fibers in the 8–10 age group compared to the 4–6 age group (+45%, *p* < 0.05) (Table 2)).

**TABLE 2:** Absolute and normalized fiber cross-sectional area, minimal ferret diameter, fiber type proportion, fiber size coefficient of variation, and relative contribution of the medial gastrocnemius muscle for the total population, boys and girls according to age groups.

	Total population				Boys				Girls			
**n**	2–4 years	4–6 years	6–8 years	8–10 years	2–4 years	4–6 years	6–8 years	8–10 years	2–4 years	4–6 years	6–8 years	8–10 years
	7	10	15	11	5	5	6	6	2	5	9	5
All fibers												
fCSA mean ± SD (µm^2^)	1,006 ± 432	1,316 ± 377	1,793 ± 370**	1,907 ± 311***^#^	887 ± 426	1,130 ± 252	1,574 ± 367	1,881 ± 209**^#^	1,305 ± 377	1,502 ± 413	1,938 ± 309	1,937 ± 430
fCSA median (IQR) (µm^2^)	1,039 (927)	1,190 (497)	1,967 (589)	1,824 (546)	658 (739)	1,118 (379)	1,552 (666)	1,883 (301)	1,305 (NA)	1,272 (781)	1,986 (404)	1,824 (826)
miniFeret mean ± SD (µm)	28.8 ± 6.0	35.9 ± 7.2	39.0 ± 4.9**	40.1 ± 3.1**	26.7 ± 5.19*	34.6 ± 7.3	36.0 ± 5.4	39.6 ± 2.0*	34.3 ± 5.1	37.2 ± 7.8	41 ± 3.5	40.7 ± 4.3
miniFeret median (IQR) (µm)	30.3 (11.3)	34.2 (11.5)	40.1 (5.2)	40.3 (4.0)	24.2 (9.6)	35.0 (12.2)	37.2 (8.6)	40.4 (3.8)	34.3 (NA)	33.4 (14.2)	40.8 (5.5)	40.1 (8.3)
CSA norm median (IQR)	2.8 (1.8)	2.3 (1.1)	2.7 (0.7)	1.9 (0.6)	1.8 (1.7)	2.0 (0.6)	2.7 (0.7)	2.0 (0.6)	3.3 (NA)	2.9 (1.2)	2.7 (0.4)	1.7 (0.10)
CV (%) median (IQR)	25.0 (6.0)	28.5 (3.8)	25.0 (3.0)	31.0 (11.0)	25.0 (5.5)	29.0 (4.0)	26.5 (16.8)	33.0 (16.3)	23.5 (NA)	27.0 (4.0)	25.0 (2.0)	29.0 (10.0)
Type I												
fCSA mean ± SD (µm^2^)	1,025 ± 454	1,410 ± 368	1,878 ± 348***	2,091 ± 331***^###^	916 ± 465	1,241 ± 278	1,745 ± 398	2,132 ± 322**^#^	1,297 ± 406	1,579 ± 436	1,967 ± 302	2,042 ± 372
fCSA median (IQR) (µm)	1,010 (956)	1,310 (454)	2,011 (420)	2,083 (207)	676 (802)	1,275 (434)	1,828 (657)	2,025 (373)	1,297 (NA)	1,398 (845)	2,083 (396)	2,111 (574)
miniFeret mean ± SD (µm)	29.2 ± 6.2	36.6 ± 7.3	40.1 ± 4.8**	42.6 ± 3.1**	27.2 ± 5.6	34.7 ± 6.9	37.9 ± 5.9	43.0 ± 2.2*	34.3 ± 5.5	38.4 ± 7.9	41.5 ± 3.5	42.1 ± 4.1
miniFeret median (IQR)	30.3 (12.1)	34.2 (10.8)	41.0 (5.9)	42.7 (2.6)	24.3 (10.4)	34.6 (10.9)	39.7 (8.5)	42.3 (3.4)	34.3 (NA)	33.8 (14.0)	41.7 (5.1)	42.7 (6.5)
CSA norm median (IQR)	2.9 (1.9)	2.5 (1.0)	2.8 (0.6)	2.2 (0.8)	1.9 (1.8)	2.3 (0.7)	3.0 (0.6)	2.2 (0.9)	3.3 (NA)	3.0 (1.2)	2.8 (0.8)	2.2 (0.7)
CV median (IQR) (%)	27.0 (6.0)	24.5 (6.5)	25.0 (5.0)	25.0 (11.0)	27.0 (5.5)	24.0 (7.0)	24.0 (10.5)	24.5 (9.0)	24.0 (NA)	25.0 (6.5)	25.0 (2.5)	25.0 (9.5)
Proportion mean ± SD (%)	66 ± 10	65 ± 6	70 ± 11	59 ± 13	61 ± 8	64 ± 7	64 ± 15	58 ± 9	77 ± 5	67 ± 4	74 ± 5	60 ± 18
Relative contribution mean ± SD (%)	67 ± 8	70 ± 6	73 ± 9	63 ± 11	64 ± 6	70 ± 6	70 ± 13	63 ± 9	76 ± 7	70 ± 6	75 ± 5	62 ± 13
Type IIa												
fCSA mean ± SD (µm^2^)	984 ± 409	1,132 ± 361	1,645 ± 448	1,829 ± 656*	849 ± 389	938 ± 218	1,313 ± 355	1,659 ± 341*	1,322 ± 283	1,326 ± 389	1,866 ± 367	2,034 ± 913
fCSA median (IQR) (µm^2^)	1,024 (857)	984 (538)	1,726 (670)	1,606 (761)	642 (676)	865 (328)	1,219 (652)	1,707 (703)	1,322 (NA)	1,184 (755)	1,849 (550)	1,568 (1,553)
miniFeret mean ± SD (µm)	28.4 ± 5.7	32.6 ± 7.6	36.3 ± 5.5	38.0 ± 6.5	26.1 ± 4.7	31.3 ± 8.7	31.9 ± 4.5	35.7 ± 3.2	34.3 ± 3.1	34.0 ± 7.1	39.3 ± 3.9	40.8 ± 8.6
miniFeret median (IQR)	29.1 (10.5)	31.2 (14.5)	37.2 (7.6)	36.5 (5.9)	24.0 (8.8)	27.2 (13.8)	31.2 (8.8)	36.8 (5.4)	34.3 (NA)	32.9 (13.7)	39.2 (6.3)	36.5 (15.2)
fCSA norm median (IQR)	2.7 (1.6)	1.9 (1.1)	2.3 (0.7)	1.7 (0.6)	1.8 (1.5)	1.7 (0.4)	2.0 (0.9)	1.8 (0.6)	3.4 (NA)	2.7 (1.2)	2.6 (0.7)	1.6 (1.9)
CV median (IQR) (%)	22.0 (8.0)	25.0 (11.0)	24.0 (6.0)	25.0 (13.0)	22.0 (7.0)	24.0 (12.5)	24.0 (12.5)	29.5 (19.3)	21.5 (NA)	26.0 (9.0)	24.0 (5.5)	24.0 (11.5)
Proportion mean ± SD (%)	28 ± 10	29 ± 4	25 ± 6	30 ± 11	32 ± 10	31 ± 5	29 ± 8	30 ± 9	19 ± 1	26 ± 2	24 ± 5	30 ± 12
Relative contribution mean ± SD (%)	27 ± 8	24 ± 3	24 ± 5	28 ± 7	30 ± 7	25 ± 3	24 ± 7	27 ± 9	19 ± 0	24 ± 3	23 ± 5	29 ± 6
Type IIx												
fCSA mean ± SD (µm^2^)	885 ± 395	1,114 ± 419	1,392 ± 403	1,330 ± 249	839 ± 423	807 ± 85	1,170 ± 388	1,329 ± 320	115 ± 309	1,359 ± 417	1,614 ± 307	1,329 ± 61
fCSA median (IQR) (µm^2^)	867 (678)	925 (644)	1,421 (733) (951)	1,394 (479)	634 (774)	809 (183)	1,158 (700)	1,436 (604)	1,115 (NA)	1,207 (778)	1,571 (572)	1,329 (NA)
miniFeret mean ± SD (µm)	26.4 ± 5.3	31.4 ± 12.0	33.4 ± 6.0	32.1 ± 3.7	25.5 ± 5.5	25.2 ± 1.1	29.9 ± 6.2	31.6 ± 4.1	34.3 ± 5.1	35.1 ± 14.4	36.8 ± 3.7	33.6 ± 2.1
miniFeret median (IQR)	26.4 (9.3)	26.2 (13.1)	34.6 (11.2)	33.3 (5.5)	22.5 (10.2)	24.6 (NA)	30.3 (11.2)	33.3 (6.9)	34.2 (NA)	30.9 (25.3)	36.8 (6.8)	33.6 (NA)
fCSA norm median (IQR)	2.4 (2.0)	1.9 (1.6)	2.1 (0.5)	1.4 (0.3)	1.9 (1.8)	1.4 (0.4)	2.1 (NA)	1.4 (0.4)	3.3 (NA)	2.9 (1.4)	2.1 (NA)	1.4 (NA)
CV median (IQR) (%)	24.0 (12.0)	24.0 (10.0)	21.0 (5.8)	27.0 (14.0)	24.0 (14.0)	23.5 (18.8)	21.0 (6.8)	27.0 (16.5)	28.0 (NA)	24.0 (13.0)	23.0 (7.5)	31.0 (NA)
Proportion mean ± SD (%)	6 ± 7	6 ± 5	5 ± 7	11 ± 11	7 ± 8	5 ± 4	8 ± 9	12 ± 7	5 ± 6	7 ± 6	3 ± 3	10 ± 15
Relative contribution mean ± SD (%)	6 ± 7	5 ± 5	4 ± 6	9 ± 10	6 ± 8	4 ± 4	6 ± 8	10 ± 6	5 ± 7	6 ± 6	2 ± 3	9 ± 14

fCSA, absolute fiber cross sectional area (µm^2^); miniFeret, minimal ferret diameter (µm); CSA norm, CSA normalised by fibula length^2^; CV, coefficient of variation. Data are shown as mean and standard deviation or indicated otherwise. Median (IQR) absolute CSA data are added for reference purposes. *p < 0.05,**p < 0.005,***p < 0.0005 compared to 2-4 age group, ^#^ *p* < 0.05,^##^ *p* < 0.005 compared to 4-6 age group. NA, not applicable.

**FIGURE 3:**
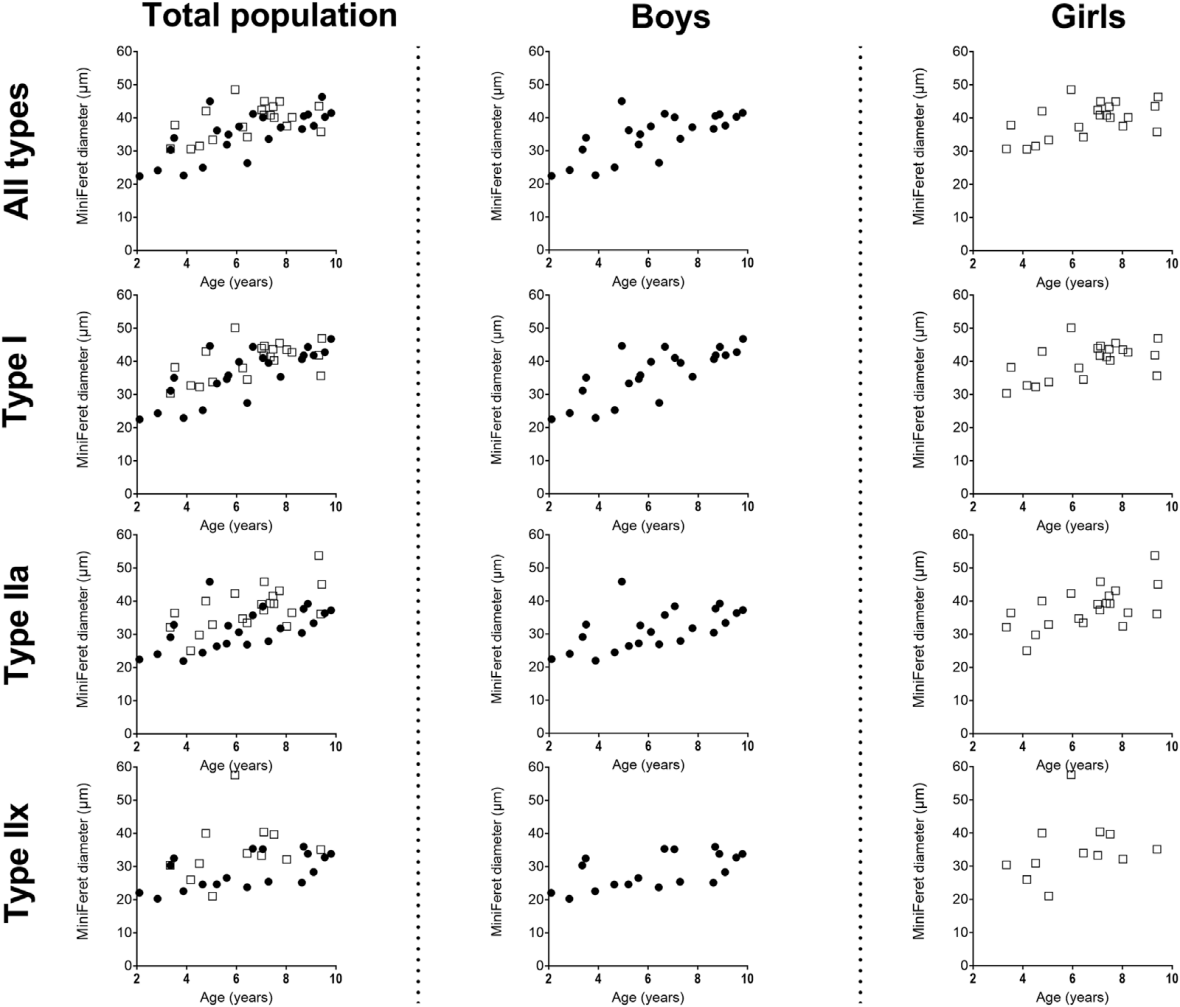
Correlation between miniFeret diameter of the medial gastrocnemius muscle and age in the total population (left panels), the boys (middle panels) and girls (right panels) for all types, type I, type IIa and type IIx fibers. *n* (2–4 years) = 7; *n* (4–6 years) = 10; *n* (6–8 years) = 15; *n* (8–10 years) = 11. Symbols: circles = boys; squares = girls.Each symbol represents individual value.

Size of the type I fiber (+104%, r = 0.76 *p* < 0.005) and type IIa (+48%, r = 0.63, *p* < 0.001) was significantly correlated with age (Figure 2B, left panel) (Table 2), while such correlation was not present for type IIx (r = 0.28, *p* = 0.122) (Figure 2B, left panel). The same pattern of changes was observed for the miniFeret diameter, where the miniFeret diameter of all fibers significantly increased with age (r = 0.63, *p* < 0.001) (Figure 3, left panel), and was significantly larger in the 6–8 and 8–10 age groups compared to the 2–4 age group (+35% and +40%, respectively) (*p* < 0.005) (Table 2). Both type I (r = 0.69, *p* < 0.001) and type IIa fiber (r = 0.55, *p* < 0.005) miniFeret diameter significantly increased with age, while the miniFeret diameter of type IIx showed a weak correlation with age (r = 0.33, *p* > 0.05) (Figure 3, left panel). Type I miniFeret diameters were significant higher in the 8–10 and 6–8 age groups compared to the 2–4 age group (+37% and +46%, respectively) (*p* < 0.005) (Table 2).

Additionally, there were no differences in the normalized fCSA of all fibers between the different age groups, as well as for each fiber type (Table 2). Inter subject variability of absolute fCSA, as reflected by the coefficient of variation (CV), ranged between 25% and 31% for all fibers and between 21% and 29% for the different fiber types, but did not significantly differ between the different age groups (Table 2).

For boys, the absolute fCSA of all fibers increased progressively with increasing age (r = 0.82, *p* < 0.001) (Figure 2B, middle panel), with the fibers in the 8–10 age group being significantly larger compared to the 2–4 age group (+112%, *p* < 0.005) and 4–6 age group (+66%, *p* < 0.05) (Table 2). Moreover, the fCSA of type I fibers and type IIa fibers strongly correlated with age (r = 0.84, *p* < 0.001 and r = 0.75, *p* < 0.001, respectively), while the fCSA of type IIx fibers slightly correlated with age (r = 0.62, *p* = 0.005) (Figure 2B, middle panel). Along the same line, the miniFeret diameter of all fibers was strongly correlated with age (r = 0.70, *p* < 0.001) (Figure 3, middle panel), and was larger in the 8–10 age group compared to the 2-4 age group (+48, *p* < 0.05) (Table 2). A significant correlation of miniFeret diameter with age was observed for type I (r = 0.77, *p* < 0.001), type IIa (r = 0.56, *p* < 0.05), and type IIx fibers (r = 0.56, *p* < 0.05) (Figure 3, middle panel). Finally, the type I miniFeret diameter was significantly higher in the 8–10 age group compared to the 2–4 age group (+58%, *p* < 0.005), while for type IIa and type IIx no differences in miniFeret diameter were seen between the different age groups (Table 2). The normalized fCSA of all fibers and of each fiber type were similar between the different age groups (Table 2). Similarly, the CV for all fibers and for each fiber type did not differ between the different age groups (Table 2).

For girls, the increase in absolute fCSA of all fiber types with increasing age was correlated with age (r = 0.64, *p* = 0.002. However, the absolute fCSA did not significantly differ between 2-4 and 8–10 age groups (+48%, *p* = 1.992) (Table 2). Moreover, also the absolute fCSA was correlated with age for type I fibers (r = 0.65, *p* = 0.002) and type IIa (r = 0.61, *p* = 0.004), while for type IIx fibers only a weak correlation was found (r = 0.38, *p* = 0.232) (Figure 2B, right panel). Absolute fCSA did not significantly differ between 2-4 and 8–10 age groups for type I (+57%, *p* = 1.608) and type IIa CSA (+54%, *p* = 2.496). The type IIa fCSA was similar between the 2–4 and 4–6 age groups and also between the 6–8 and the 8–10 age groups, but the latter was higher compared to the younger age groups (Table 2). Finally, the absolute fCSA of the type IIx fiber tended to be slightly larger between the 4-6 age groups and remained stable in the other age groups (Table 2). The miniFeret diameter of all fibers was moderately correlated with age (r = 0.55, *p* = 0.010) (Figure 3, right panel), and did not differ between the different age groups (Table 2). Moreover, the miniFeret diameter of type I fibers moderately correlated with age (r = 0.55, *p* = 0.011), as did the miniFeret diameter for type IIa fibers (r = 0.59, *p* = 0.005), while a weak correlation was found for the miniFeret diameter of type IIx fibers (r = 0.26, *p* = 0.419). Finally, there were no differences in miniFeret diameter between the different age groups, irrespective of the fiber type (Figure 3; Table 2).

No differences were found in the normalized fCSA of all fibers and each fiber type between the different age groups (Table 2). The average CV did not differ between the different age groups, irrespective of the fiber type (Table 2).

For all age groups, the absolute fCSA did not differ between girls and boys. However, the absolute fCSA were higher in girls at young ages compared with boys, which was a phenomenon that disappeared in the 8–10 age group. The normalized fCSA and the CV were similar between boys and girls for all fibers and each fiber type (Table 2).

#### 3.2.2 Fiber type size distribution frequency

**FIGURE 4:**
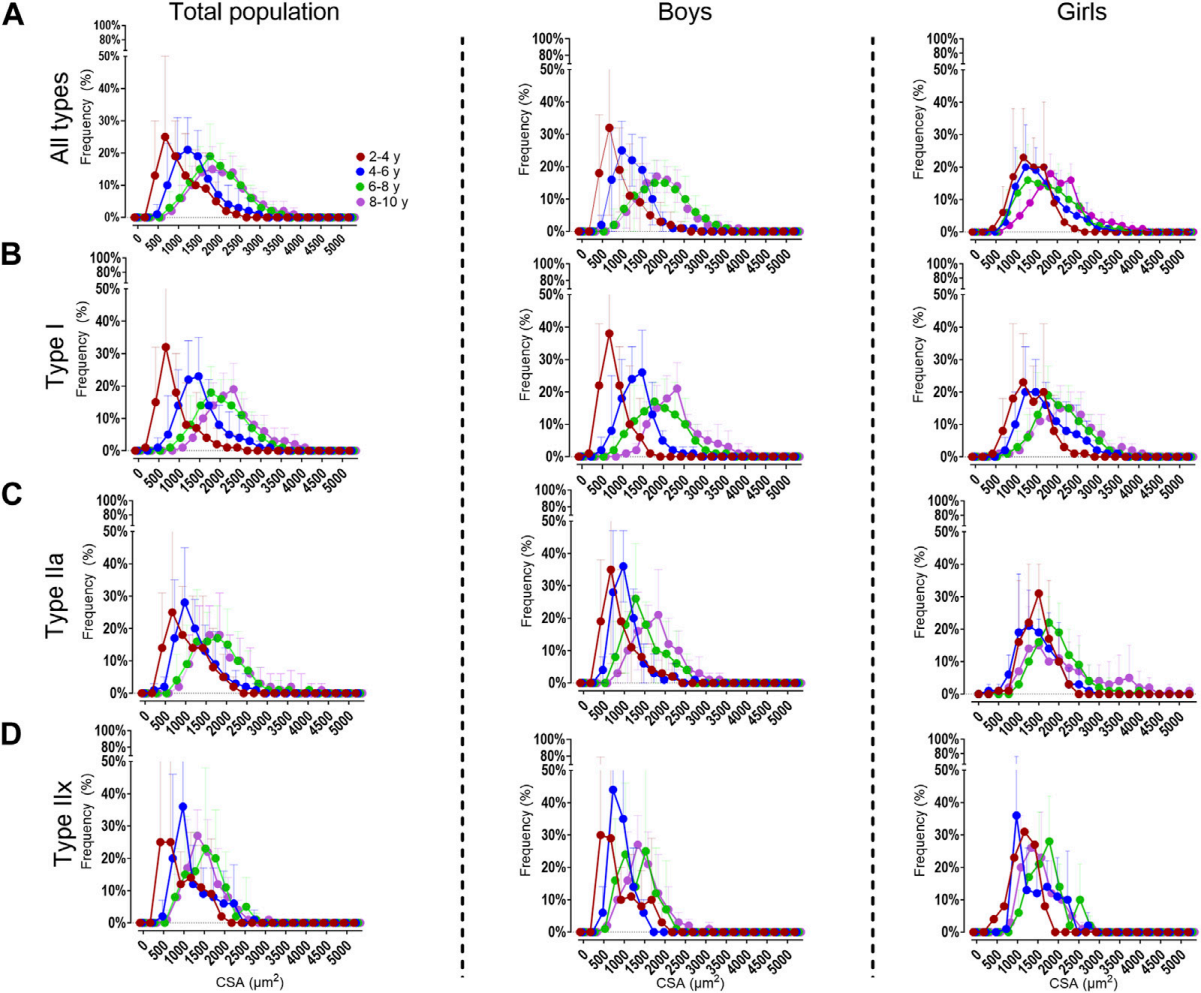
Distribution frequency of the absolute fiber cross-sectional area for all fiber types **(A)**, the type I **(B)**, type IIa **(C)** and type IIx **(D)** fibers in the medial gastrocnemius muscle for the total population (left panels), boys (middle panels) and girls (right panels) according to age groups. Data are shown as mean and standard deviation. N (2–4 years) = 7; *n* (4–6 years) = 10; *n* (6–8y ears) = 15; *n* (8–10 years) = 11.

For the total population, the 2–4 age group had a larger proportion of smaller fibers (i.e., between 0 and 750 μm^2^ bins, 38% ± 43%) compared to the 6–8 and in the 8–10 age groups (3% ± 5% and 2% ± 6%, respectively *p* < 0.001) (Figure 4A, left panels). These findings were observed for type I and type IIa fibers (Figures 4B, C, left panels). Also the type IIx fibers were smaller (i.e., between 0 and 1,000 μm^2^ bins) in the 2–4 age group compared to the 6–8 and 8–10 age group (*p* < 0.001).

For boys, the 2–4 age group had smaller fibers (i.e. 0–750 μm^2^ bins 51% ± 46%) compared to the 6–8 and 8–10 age groups (6% ± 8% and 2% ± 8%, respectively *p* < 0.001). The same was observed for the type IIa fibers. For the type I fibers, the fibers were smaller (i.e. 0-1,000 μm^2^ bins) in the 2–4 age group (67% ± 75%), compared to the 6–8 and 8–10 age groups (9% ± 28% and 8% ± 16%, respectively *p* < 0.001) (Figures 4A–C, middle panels).

For girls, the fibers were smaller in the 2–4 age group (i.e 0-1,000 μm^2^ bins 24% ± 31%) compared the 8–10 age groups (6% ± 6% *p* < 0.001) (Figure 4A, right panels). The same trend was seen for the type I (Figure 4B, right panels) and type IIa (Figure 4C, right panels). The distribution frequency of the fCSA of type IIx fibers indicated that there were no differences between the age groups (Figure 4D, right panels).

Moreover, the proportion of all fibers were larger in girls at younger age compared to boys at the same age (*p* < 0.001). However, these differences disappeared in the older age groups. The same observation was made for the type I and type IIa fibers (*p* < 0.001).

#### 3.2.3 Fiber type proportion

For the total population, the fiber proportion averaged 65% ± 11% for the type I, 28% ± 8% for the type IIa and 7% ± 8% the type IIx with no differences between the different age groups (Table 2). Overall, the same proportion was found when only investigating boys, with the type I fiber proportion averaging 62% ± 10%, and that of the type IIa and IIx 31% ± 8% and 7% ± 7%, respectively. There were no differences in fiber type proportion between the different age groups (Table 2). Similarly, the fiber proportion for girls averaged 69% ± 8% for type I, 25% ± 3% for type IIa and 6% ± 3% for type IIx. The proportion of each fiber type did not differ between the different age groups (Table 2). Finally, fiber type proportion for all fibers and each fiber type did not significantly differ between boys and girls whatever the age group.

#### 3.2.4 Relative fiber contribution to total surface

For the total population, the relative contribution of the type I fiber to the total surface was on average 69% ± 9% while it was 25% ± 6% for the type IIa and 6% ± 7% for the type IIx fibers. There were no differences in the average relative contribution of each fiber type to the total surface between the different age groups (Table 2). In boys, the type I fiber relative contribution to the total surface was 67% ± 9% and that of type IIa and IIx was 26% ± 7% and 7% ± 7%, respectively. The relative fiber contribution did not differ between the age groups for either type I, type IIa or type IIx fibers (Table 2). In girls, the relative fiber contribution to the total surface did not change between the different age groups (Table 2), with average values being 71% ± 9% for type I, 24% ± 5% for type IIa and 5% ± 8% for type IIx.

Finally, the relative fiber contribution to the total surface did not significantly differ between boys and girls for all age groups. However, in the 2–4 age group, type I relative contribution was higher (+19%, *p* = 0.190) and type IIa relative contribution was lower (−37%, *p* = 0.095) in girls compared to boys, but failed to reach statistical significance.

### 3.3 Capillarization

**FIGURE 5:**
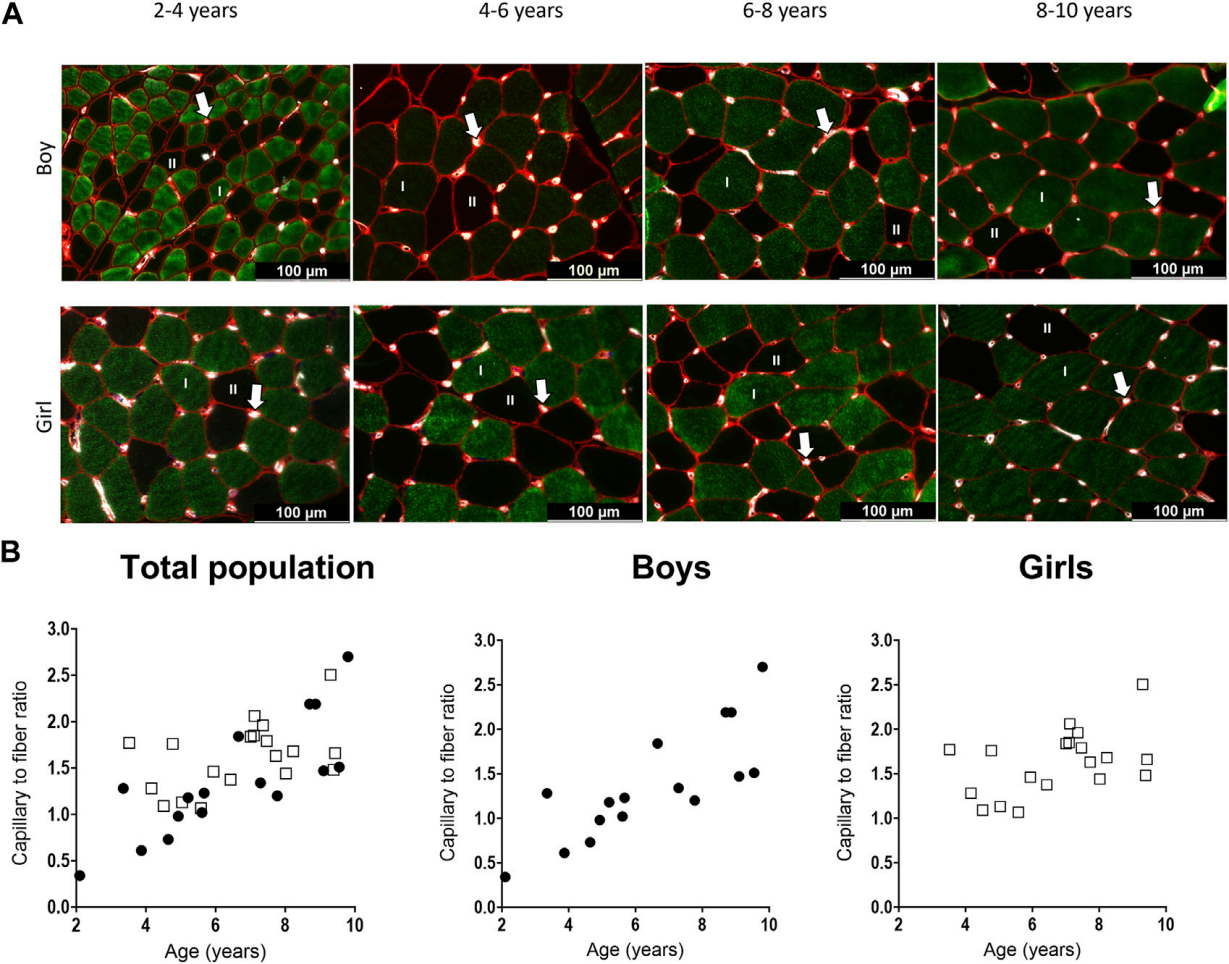
**(A)** Representative examples of the capillary staining in the medial gastrocnemius muscle of boys (upper panels) aged 2 years, 6 years, 7 years, 9 years, and in girls (lower panels) aged 4 years, 5 years, 7 years, and 9 years. Type I fibers are in green (I), type II fibers are in black (II), laminin surrounding the cells is in red and capillaries are the white spots, indicated with an arrow. Scale represents 100 μm. **(B)** Correlation between capillary to fiber ratio and age for the total population (left), the boys (middle and the girls (right). *n* (2–4 years) = 4; *n* (4–6 years) = 10; *n* (6–8 years) = 10; *n* (8–10 years) = 10. Symbols: circles = boys; squares = girls. Each symbol represents individual value.

Representative examples of capillaries stained with CD31 in combination with MHC staining for type I in the MG muscle are depicted in Figure 5 for boys and girls according to age range. For the assessment of the C/F and CFD, we counted on average 140 ± 77 fibers (for boys:157 ± 96 fibers and for girls 138 ± 56 fibers). The number of counted fibers ranged from 77–424 for boys, and from 100–310 for girls.

For the total population, the C/F increased gradually with age (Table 3) (Figure 5B, left panel). There was a strong positive correlation between the C/F and age (r = 0.63, *p* < 0.0001). C/F was significantly higher in the 8–10 (1.6 fold increase, *p* < 0.05) and 6–8 (1.4-fold increase, *p* < 0.05) age groups compared to the 4-6 age group (Table 3). Also for boys, there was a positive and strong correlation between the capillary to fiber ratio and age (r = 0.83, *p* < 0.001) (Figure 5B, middle panel). In addition, the 8–10 age group had a significant higher C/F compared to the 2–4 and 4–6 age groups (2.7 and 1.9 fold increase, respectively *p* < 0.05) (Table 3). By contrast, in girls, the correlation between C/F and age was weak (r = 0.374, *p* = 0.115) (Figure 5B, right panel) and C/F was significantly increased but solely in the 6–8 age group compared to the 4–6 age group (1.3 fold increase, *p* < 0.05) (Table 3). Finally, there were no differences in C/F ratio between boys and girls irrespective of the age groups, and regardless of fiber type.

**TABLE 3:** Capillary to fiber ratio, capillary fiber density for type I and II fibers, capillary domains, heterogeneity index and the Capillary to fiber perimeter (CFPE) index for type I and type II of the medial gastrocnemius muscle for the total population, the boys and the girls according to age groups.

	Capillary to fiber ratio	Capillary Fiber Density type I fibers (Capillaries/mm^2^)	Capillary Fiber Density type II fibers (Capillaries/mm^2^)	Capillary domains (µm^2^)	Heterogeneity Index (LogSD)	Capillary-to-fiber perimeter exchange index type I ( $\left.1000^{-1}\right)$	Capillary-to-fiber perimeter exchange index type II ( $\left.1000^{-1}\right)$
Total population							
2–4 years (*n* = 4)	1.00 ± 0.65	551 ± 124	543 ± 137	1,933 ± 380	0.19 (0.02)	4.37 ± 1.57	3.85 ± 1.21
4–6 years (*n* = 11)	1.18 ± 0.27*^#^	514 ± 70	504 ± 90	2,120 ± 311	0.18 (0.05)	4.68 ± 0.65	3.93 ± 0.61
6–8 years (*n* = 10)	1.69 ± 0.29	489 ± 62	475 ± 75	2,172 ± 290	0.19 (0.04)	5.11 ± 0.50	4.47 ± 0.70
8–10 years (*n* = 10)	1.88 ± 0.47	491 ± 89	484 ± 79	2,186 ± 428	0.17 (0.02)	5.76 ± 1.0	5.00 ± 1.09
Boys							
2–4 years (*n* = 3)	0.74 ± 0.48^#^	558 ± 151	563 ± 160	1,931 ± 465	0.19 (NA)	4.16 ± 1.86	3.76 ± 1.46
4–6 years (*n* = 5)	1.03 ± 0.20^#^	522 ± 92	508 ± 115	2,153 ± 417	0.18 (0.05)	4.45 ± 0.82	3.58 ± 0.57
6–8 years (*n* = 3)	1.46 ± 0.34	494 ± 54	456 ± 61	2,144 ± 268	0.21 (NA)	5.00 ± 0.83	3.76 ± 0.21
8–10 years (*n* = 5)	2.01 ± 0.52	535 ± 46	533 ± 51	1,954 ± 248	0.18 (0.02)	6.33 ± 0.67	5.18 ± 0.65
Girls							
2–4 years (*n* = 1)	1.77±(NA)	528±(NA)	482±(NA)	1,938±(NA)	0.21 (NA)	4.98±(NA)	4.13±(NA)
4–6 years (*n* = 6)	1.30 ± 0.27*	508 ± 58	500 ± 74	2,092 ± 227	0.16 (0.04)	4.87 ± 0.47	4.23 ± 0.49
6–8 years (*n* = 7)	1.79 ± 0.23	487 ± 69	483 ± 83	2,184 ± 318	0.19 (0.04)	5.16 ± 0.37	4.77 ± 0.60
8–10 years (*n* = 5)	1.75 ± 0.43	449 ± 106	436 ± 76	2,379 ± 466	0.17 (0.03)	5.18 ± 1.0	4.80 ± 1.47

Data shown as mean and standard deviation, except for the heterogeneity index which is shown as median (interquartile range). *p < 0.05 compared to 6-8 age group, ^#^ *p* < 0.05 compared to 8–10 age group. NA, not applicable.

For the total population, the CFPE index correlated moderately with age for both type I (r = 0.41, *p* = 0.015) and type II fibers (r = 0.35, *p* = 0.042) (Table 3). There were no differences in CFPE index between the different age groups (Table 3). For boys, there was a stronger correlation between the CFPE index and age for both type I (r = 0.65, *p* < 0.05) and type II fibers (r = 0.55, *p* < 0.05). The CFPE index for type I was 1.3 fold higher in the 8–10 age group compared to the 2–4 age group, however this failed to reach significance, and the same was seen for the CFPE index of type II fibers (Table 3). Moreover, for girls, the correlation between the CFPE index and age was negligible for both type I (r = −0.047, *p* = 0.847) and type II fibers (r = 0.030, *p* = 0.904) (Table 3). Furthermore, there were no differences in CFPE index between the different age groups. As well as, between boys and girls regardless of fiber type or age group (Table 3).

The CFD for type I and II fibers was similar between all age groups for the total population, and also for boys and girls (Table 3). No correlations were found between CFD of type I and age for the total population (r = −0.225, *p* = 0.193), and also for boys (r = −0.115, *p* = 0.672) and girls (r = −0.458, *p* = 0.049). Similarly, there were no relationships between the CFD of type II fibers and age for the total population (r = −0.197, *p* = 0.258), for boys (r = −0.100, *p* = 0.713) and for girls (r = −0.432, *p* = 0.065). For the total population, but also separately for boys and girls, the capillary domains and the heterogeneity index were similar in the different age groups (Table 3).

### 3.4 Number of satellite cells to fiber type

**FIGURE 6:**
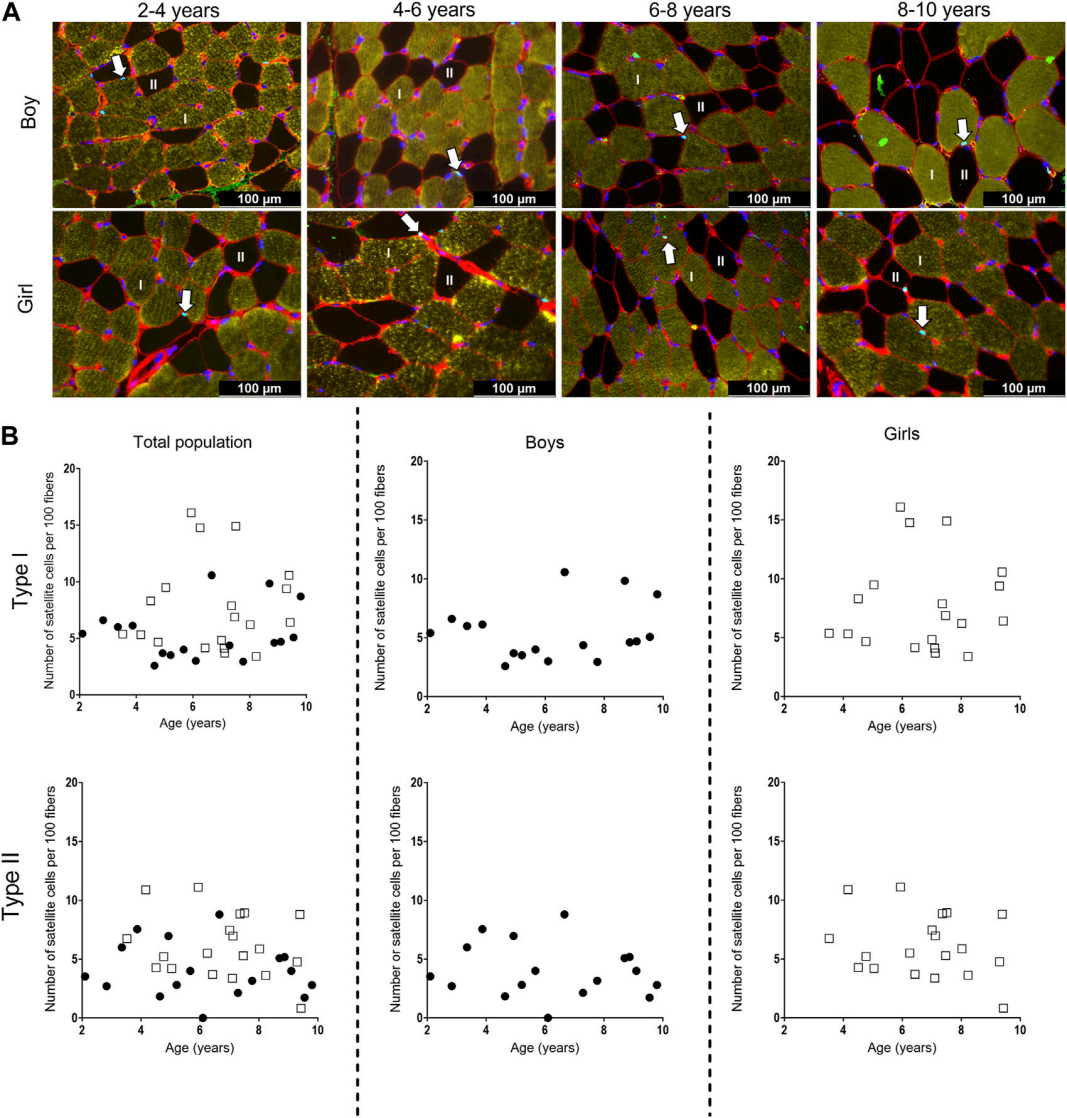
**(A)** Representative examples of the satellite cell staining in the medial gastrocnemius muscle of boys (upper panel) aged 3 years, 6 years, 7 years, 10 years, and girls (lower panels) aged 3 years, 6 years, 7 years, and 8 years. Type I fibers are in yellow (I), type II fibers are in black and (II), laminin surrounding the cells is in red, nuclei are stained in dark blue and satellite cells appear as light blue spots and are indicated with an arrow. Scale represents 100 µm. **(B)** Correlation between satellite cell number per 100 fibers and age for type I (upper panels) and type II (lower panels) fibers in the medial gastrocnemius muscle for the total population (left panels), the boys (middle panels) and the girls (right panels) according to age. Symbols: circles: boys; squares girls. Each symbol represents individual value.

Representative examples of the SC stained with PAX7 in combination with MHC staining for type I and II fibers types in the MG muscle are depicted in Figure 6A for boys and girls according to age range. For the assessment of the number of SC per 100 fibers, we counted on average 534 ± 314 fibers (for boys: 498 ± 260 fibers and for girls 574 ± 363 fibers). The number of counted fibers ranged from 196–1,049 for boys, and from 204–1,240 for girls.

For the total population, the number of SC did not differ between the different age groups for both type I and type II fibers. Moreover, there was a high variability in the number of SC in each age group, regardless of the fiber type (Figure 6B, left panels). Along the same line, no differences in the number of SC were seen between the different age groups for both boys and girls, nor between boys and girls (Figure 6B, middle and right panels). However, the number of SC for both type I and type II fibers was highly variable in boys and girls at all age ranges (Table 4). No relationships were found between the number of SC per 100 type I fibers and age for the total population (r = 0.133, *p* = 0.440), boys (r = 0.071, *p* = 0.786) and girls (r = 0.058, *p* = 0.814). Similarly, no correlations were present between the number of SC per 100 type II fibers and age (Total population: r = −0.153, *p* = 0.374, boys: r = −0.161, *p* = 0.538 and girls: r = −0.182, *p* = 0.455).

**TABLE 4:** Number of satellite cells per 100 type I and type II fibers of the medial gastrocnemius muscle for the total population, boys and girls according to the age groups.

	Number SC/100 type I fibers	Number SC/100 type II fibers
Total population		
2–4 years (*n* = 5)	6 (0.98)	6 (4.03)
4–6 years (*n* = 9)	5 (5.30)	4 (5.53)
6–8 years (*n* = 12)	5 (6.43)	5 (5.29)
8–10 years (*n* = 10)	6 (4.83)	4 (2.82)
Boys		
2–4 years (*n* = 4)	6 (0.93)	5 (4.25)
4–6 years (*n* = 4)	4 (1.11)	3 (4.15)
6–8 years (*n* = 4)	4 (6.39)	3 (7.01)
8–10 years (*n* = 5)	5 (4.62)	4 (2.87)
Girls		
2–4 years (*n* = 1)	5 (NA)	7 (NA)
4–6 years (*n* = 5)	8 (7.80)	5 (6.76)
6–8 years (*n* = 8)	6 (8.93)	6 (4.40)
8–10 years (*n* = 5)	6 (5.18)	5 (5.11)

Data shown as median and interquartile range. NA, not applicable.

## 4 Discussion

This is the first study examining fiber type size and proportion, SC and capillarization in MG muscle of young TD children according to age and gender. This study showed that absolute fCSA increased with age, but mainly in boys since girls had larger fiber sizes at young ages compared to boys. However, at the age of 8–10 years, fiber sizes were similar between boys and girls. This study also revealed that fiber size variability was present at all ages for both genders but was of same magnitude across age group and gender. Similarly, fiber type proportion, number of SC and capillarization were of same magnitude, while C/F was strongly correlated with age in boys but weakly in girls. So, this study provides reference data of common histological measures in the MG muscle of young healthy children. Our data indicated that some histological features in the MG muscle differ between boys and girls especially at young ages. This should be taken into account when comparing histological data from children of both genders.

The current study showed a progressive increase in body height, body weight, tibia and fibula length with age. These findings are in agreement with previous data showing that tibia length and body weight increased with age of children aged 5–12 years, in whom no gender related differences were observed (Bénard et al., 2011). Further, the Center for disease control and prevention has growth charts of both body height and body weight for boys and girls aged 2–10 years, which are combined data from several national American surveys. The body height curve and body weight for boys as well as for girls started around 85 cm at 2 years of age and goes up to around 135 cm at 10 years, which is in agreement with our data. Moreover, the growth curves of the Center for disease control and prevention indicated that the growth of boys and girls was the same, which is in accordance with our findings (Centers for Disease Control and PreventionNational Center for Health Statistics, 2017). To the best of our knowledge, data on fibula length are not yet reported for young healthy children.

In the present study, absolute fCSA area and the miniFeret diameter of the MG increased with increasing age. Our data confirms the steady increase in fiber area of the MG reported on *postmortem* material in children aged 2 months till 18 years who died either from road accident or from acute infective illness (*n* = 23) (Aherne et al., 1971). To the best of our knowledge, additional literature on the MG fCSA with age is not available, since previous studies mainly focused on other lower limb muscles or on upper limb muscles. Indeed, in an autopsy study on 113 subjects who died from accidents and aged 1 week till 20 years, an increase in fiber diameter for both type I and type II fibers with increasing age was observed in the vastus lateralis and deltoid muscles (Oertel, 1988). The extent of the type I fCSA increases between the 2–4 and 8–10 years age group in our study (+104%) was in the same range than the increase reported in fiber diameter between the age group 1.5–3 years and 6–10 years for the vastus lateralis muscle (+126%) and for the deltoid muscle (+143%) (Oertel, 1988). In line with our data on fCSA and miniFeret diameter in the gastrocnemius, the fiber diameter for both type I and type II fibers were shown to increase with age in the brachial biceps of children aged 3 till 10 years and also in the quadriceps muscle of children aged 2 till 12 years (Sallum et al., 2013). Progressive increase in fCSA with age was also observed in thoraco-abdominal muscles in children aged 5 days to 15 years and in rectus abdominis, pectoralis major, and sartorius muscles of children aged less than 18 years (Delhaas et al., 2013; Verdijk et al., 2014). Our study also showed a greater increase in fCSA and miniFeret diameter of the MG in young boys starting from the age of 2 years, compared to young girls, an effect which disappeared when boys were getting older. Our data strongly suggest that population needs to be matched for both gender and age when comparing data from young boys and girls in this age range. However, Oertel et al. found no gender related differences in the fiber diameters of the vastus lateralis and deltoid muscles in children before the age of 15 years (Oertel, 1988). However, their data should be interpreted with caution as they refer to data from autopsy muscle samples with part of them being issued from ill children compared to our study on fresh muscle samples in healthy children. We therefore believe that the gender differences in fCSA seen with age in the current study are reflecting a true effect of difference in fiber growth with age in the MG of healthy young children.

In this study, fiber size variability was present in the MG of young healthy children but it was similar between the different fiber types. Our data of fiber size variability corroborate the findings in the vastus lateralis muscle in children aged 9–11 years showing a coefficient of variation of 28% ± 6 for boys and 30% ± 8 for girls in type I fibers and of 21% ± 5 (boys) and 23% ± 7 (girls) for type IIa fibers (Esbjörnsson et al., 2022). In the peroneus brevis muscle, a CV of 17% for type I and II fibers was reported in samples issued from pathology data of children aged 5–13 years (Rose et al., 1994). These data, which are lower than the values reported for lower limb muscles, suggest that although fiber size variability is present in healthy children, its magnitude is likely to differ between muscle type. There are currently no data reporting fiber size variability with age according to gender and fiber type especially at young ages. Our data showed that fiber size variability in the MG muscle remains stable with increasing age, and was similar irrespective of the fiber type and gender. In summary, this study underlines that fiber size variability is present in the MG muscle of healthy young children but remains of the same magnitude with age and is independent of gender and fiber type. Next to fCSA, data on fiber size variability should be considered when assessing fiber dimension as it may better reflect changes in fiber size than average value of fCSA.

As far as we are aware, this is the first study reporting data on fCSA distribution frequency with age in healthy and (very) young children. Our data showed more fibers with smaller size in the younger age groups. In fact, young boys had more fibers with smaller size compared to young girls and this was observed for each fiber type in boys. These data underline that matching for age and gender should be considered in young children. Especially, when data on fCSA distribution frequency of the MG would be used as reference values.

Until this study, there were no data on fiber type proportion of the MG muscle in healthy children according to age and gender. Our data indicated that the MG is mainly composed of type I fiber in young children and that the fiber type proportion remained stable with increasing age, as well as between gender at least up to 10 years. Unchanged fiber type proportion with age was previously reported in children for the vastus lateralis muscle (Oertel, 1988) and deltoid muscles (Oertel, 1988) as well as for the rectus abdominis, pectoralis major, or sartorius muscles (Verdijk et al., 2014). Furthermore, for the latter muscle types, no gender related differences in fiber type proportion was observed (Kriketos et al., 1997). Our data together with the findings from previous literature suggest that fiber type proportion of a given muscle is not changing with increasing age and is not affected by gender in healthy children up to 10 years.

So far, only one study has investigated muscle capillarization in healthy young children according to age. In that study, an increase in C/F with age was reported in the brachial biceps and quadriceps muscles of children aged 2–12 years (Sallum et al., 2013). Our data revealed that this was also the case in the MG muscle for the total population. But, while C/F increased with age in boys, in whom a strong positive correlation between capillary to fiber ratio with age was found, this was not the case in girls. Although surprising at first sight, these data fit with our findings on fiber size showing larger fCSA at younger age in girls compared to boys and thus a moderate increase in fCSA with age in girls compared to boys. Keeping in mind that the number of capillaries is related to fiber size, the larger fibers as seen in the MG of young girls are expected to be surrounded by a larger number of capillaries (Zoladz et al., 2005; Hendrickse and Degens, 2019), explaining thereby why the C/F did not increase with age in girls. On the other hand, because the fCSA in boys at young age were small and their size increased substantially with age, the increased C/F in the MG muscle in boys with increasing age is compatible with the progressive increase in fiber size seen in boys with age. Nevertheless, these findings concerning C/F in girls need to be interpreted with caution given the low sample size in the younger age groups especially the 2–4 age group where only one girl was included. At least, our data suggest that the oxygenation, nutrient delivery and pro-angiogenic compounds are maintained with age, as well as the mitochondrial volume density (Barnouin et al., 2017) which indicates aerobic capacity and maximal oxygen uptake (Patel et al., 2009).

In this study, we also showed that the CFD, which is used to determine the total oxygen transport and diffuse distance into the muscle (Chen and Diana, 1984; McGuire and Secomb, 2003), remained stable with age whatever the gender. To the best of our knowledge, there are no data on CFD according to age in any skeletal muscle of healthy young children. Our data indicated that the diffusion distance of oxygen did not change with age and with increase in fCSA, which is probably due to the fact that the number of capillaries per fiber increased. This means that more capillaries are available and therefore the diffusion distance of oxygen to the mitochondria is not altered.

Furthermore, in our study, the capillary domain, which is an indication of the tissue supply area surrounding a capillary (Barnouin et al., 2017), did not change with age or gender, indicating that the overall function of one capillary is not altered with increasing age or with gender. Most likely, capillary environment adapts through angiogenesis making that the function of one capillary remains stable with increasing age.

Finally, besides the number of capillaries per fiber and fiber area, tissue oxygenation also depends on the way capillaries are distributed in the muscle. The heterogeneity index was therefore assessed in the current study, as it represents the distribution of the capillary spacing. It is important to keep in mind that a very heterogenous distribution of the capillaries has a negative impact on the muscle capillarization (Degens et al., 2006; Barnouin et al., 2017). Such a parameter has never been investigated in the muscle of young children according to age. In the current study, heterogeneity index remained stable with age and was not affected by gender. Since the heterogeneity index in the MG muscle did not change with age, while the number of capillaries per fiber increased, it is most likely that capillaries were not randomly organized in the muscle but added in a rather controlled manner to ensure optimal tissue capillarization (Degens et al., 2006). Obviously, this is needed to maintain appropriate functioning of the muscle during children growth.

It is well known that SC play an essential role in normal myogenesis of childhood growth, ensuring hypertrophy of the muscle fibers, enabling muscle growth according to age (Frank, 2012; Pallafacchina et al., 2013; Carlson, 2022). In fact, during growth, muscle fibers increase in CSA but also in length. It is shown that in the MG, fiber length increases by 5% per year between the age of 5–12 years (Bénard et al., 2011). Childhood development requires thus a continuous activation, proliferation and differentiation of the SC into new myofibers, but whether this is associated with a certain decline of the SC pool is not clear (Frank, 2012; Pallafacchina et al., 2013; Carlson, 2022). There are, in fact, very little data on the number of SC in the muscle of healthy young children according to age and gender. In addition, inconsistent results have been reported so far. In lower limb muscles, the number of SC per muscle fiber was reported to slightly increase in children within the 2–9 years age range (Kottlors and Kirschner, 2010) but due to the low sample size of these control samples (only 5 children), no statistics were performed and it is unlikely that this increase would have reached statistical significance. In addition, the poor distribution of these five controls over the 2-9 age range (1 sample at 2, 3, 4 years, and 2 samples at 9 years) makes it difficult to draw firm conclusion regarding the SC content according to age as there are no data between 4 and 9 years (Kottlors and Kirschner, 2010). In a study on pooled data on the rectus abdominis, pectoralis major, and sartorius muscles, a modest increase in the number of SC per type I and type II muscle fibers was found in children from 2 to 18 years but this increase was not observed in children aged 2–10 years and was actually mainly due to higher number of SC in the children aged 15–18 years (Verdijk et al., 2014). Finally, a steep decline in SC density in pooled data of thoraco-abdominal muscles was shown in the first month of life while it remained stable afterwards with increasing age with no differences between gender (Delhaas et al., 2013). In our study, the number of SC per 100 fibers in the MG did not differ with increasing age regardless of fiber type and gender. The absolute values we found (3–16/100 type I and 0 to 11/100 type II fibers) were, however, in agreement with previous data on lower limb muscles (0.05–0.12 SC per fiber) (Verdijk et al., 2014). In addition, our data underline that the number of SC is highly variable for a given age and also between children with different age. Similar variability in SC content according to age was also observed in previous literature (Delhaas et al., 2013; Verdijk et al., 2014). Altogether, our data suggest that the number of SC in the MG muscle remained constant with age in healthy children between the age 2–10 years, with no differences between type I and type II fiber SC content. Our data also confirmed that a high variability in the number of SC according to age is present in the muscle of healthy growing children, suggesting that this parameter might be difficult to use as reference value parameter.

This study present some limitations that need to be mentioned. First, the cross-sectional design of the study made it not possible to follow muscle growth of the same child over time. Second, the method used to assess capillarization based on 2D muscle cross-sections may not adequately represents the 3D structure and distribution of the muscle capillarization (Zeller-Plumhoff et al., 2017). However, although not fully perfect, this method which is accessible to most researchers has been extensively used to evaluate capillarization. We therefore believe that the reference values of the current study may be helpful to the researchers using this way of assessing capillarization. Thirdly, this current study did not assess the presence of hybrid fibers, although it would be of value. However, the major problem is that it remains difficult to determine sample size and amount of hybrid fibers that need to be counted to provide accurate reference data on these fibers. Finally, it is important to keep in mind that the data of the current study refer to reference values for biopsies that have been taken in the muscle belly of the gastrocnemius and they might not be appropriate as reference values for biopsies taken close to the tendon, where regional differences in fiber composition is expected.

## 5 Conclusion

This is the first study providing data on fCSA, fiber type proportion, number of SC and capillarization in the MG of the same child according to age and gender. Overall, these data provide reference values of commonly used histological measures of the MG for normally growing children between 2 and 10 years. This normative dataset may be used to evaluate the impact of disease on muscle histology as well as the efficacy of therapeutic strategies.

## Data Availability

The original contributions presented in the study are included in the article/Supplementary material, further inquiries can be directed to the corresponding author.

## References

[B1] Aherne W. Ayyar D. R. Clarke P. A. Walton J. N. (1971). Muscle fibre size in normal infants, children and adolescents. An autopsy study. J. Neurological Sci. 14 (2), 171–182. 10.1016/0022-510x(71)90085-2 5112209

[B2] Ballak S. B. Yap M. H. Harding P. J. Degens H. (2016). Validation of a new semi-automated technique to evaluate muscle capillarization. Adv. Exp. Med. Biol. 876, 87–93. 10.1007/978-1-4939-3023-4_11 26782199

[B3] Bankolé L. C. Feasson L. Ponsot E. Kadi F. (2013). Fibre type-specific satellite cell content in two models of muscle disease. Histopathology 63 (6), 826–832. 10.1111/his.12231 24111647

[B4] Barnouin Y. McPhee J. S. Butler-Browne G. Bosutti A. De Vito G. Jones D. A. (2017). Coupling between skeletal muscle fiber size and capillarization is maintained during healthy aging. J. Cachexia, Sarcopenia Muscle 8, 647–659. 10.1002/jcsm.12194 28382740 PMC5566646

[B5] Bénard M. R. Harlaar J. Becher J. G. Huijing P. A. Jaspers R. T. (2011). Effects of growth on geometry of gastrocnemius muscle in children: a three-dimensional ultrasound analysis. J. Anat. 219 (3), 388–402. 10.1111/j.1469-7580.2011.01402.x 21635250 PMC3171775

[B6] Bloemberg D. Quadrilatero J. (2012). Rapid determination of myosin heavy chain expression in rat, mouse, and human skeletal muscle using multicolor immunofluorescence analysis. PLoS ONE 7 (4), 35273. 10.1371/journal.pone.0035273 PMC3329435 22530000

[B7] Carlson B. (2022). “Normal muscle growth,” in Muscle biology (Amsterdam, Netherlands: Elsevier), 57–75. 10.1016/b978-0-12-820278-4.00003-7

[B8] Carlson M. E. Suetta C. Conboy M. J. Aagaard P. Mackey A. Kjaer M. (2009). Molecular aging and rejuvenation of human muscle stem cells. EMBO Mol. Med. 1 (8–9), 381–391. 10.1002/emmm.200900045 20049743 PMC2875071

[B9] Centers for Disease Control and Prevention, National Center for Health Statistics (2017). CDC growth charts. https://www.cdc.gov/growthcharts/clinical_charts.htm.

[B10] Chen I. I. H. Diana J. N. (1984). Mathematical modeling of capillary density. J. Theor. Biol. 108 (2), 221–225. 10.1016/s0022-5193(84)80068-5 6748689

[B11] Corvelyn M. De Beukelaer N. Duelen R. Deschrevel J. Van Campenhout A. Prinsen S. (2020). Muscle microbiopsy to delineate stem cell involvement in young patients: a novel approach for children with cerebral palsy. Front. Physiology 11 (945), 945. 10.3389/fphys.2020.00945 PMC7424076 32848872

[B12] Degens H. Deveci D. Botto-Van Bemden A. Hoofd L. J. C. Egginton S. (2006). Maintenance of heterogeneity of capillary spacing is essential for adequate oxygenation in the soleus muscle of the growing rat. Microcirculation 13 (6), 467–476. 10.1080/10739680600776286 16864413

[B13] Delhaas T. Sander F. Van der Meer T. Schaart G. Degens H. Drost M. R. (2013). Steep increase in myonuclear domain size during infancy. Anat. Rec. 296 (2), 192–197. 10.1002/ar.22631 23213045

[B14] Domenighetti A. A. Margie A. M. Pichika R. Sibley L. A. Zhao L. Chambers H. G. (2018). Loss of myogenic potential and fusion capacity of muscle stem cells isolated from contractured muscle in children with cerebral palsy. Am. J. Physiol. Cell Physiol. 315 (2), 247–257. 10.1152/ajpcell.00351.2017 PMC6139501 29694232

[B15] Esbjörnsson M. Norman B. Dahlström M. Gierup J. Jansson E. (2022). Metabolic and morphological profile in skeletal muscle of healthy boys and girls. Physiol. Rep. 10 (16), e15414. 10.14814/phy2.15414 35986491 PMC9391602

[B16] Esbjörnsson M. E. Dahlström M. S. Gierup J. W. Jansson E.Ch (2021). Muscle fiber size in healthy children and adults in relation to sex and fiber types. Muscle Nerve 63 (4), 586–592. 10.1002/mus.27151 33347630 PMC8048954

[B17] Forcina L. Miano C. Pelosi L. Musarò A. (2019). An overview about the biology of skeletal muscle satellite cells. Curr. Genomics 20 (1), 24–37. 10.2174/1389202920666190116094736 31015789 PMC6446479

[B18] Frank M. (2012). “Encyclopedia of exercise medicine in Health and disease,” in Encyclopedia of exercise medicine in Health and disease (Berlin Heidelberg: Springer). 10.1007/978-3-540-29807-6

[B19] Hendrickse P. Degens H. (2019). The role of the microcirculation in muscle function and plasticity. J. Muscle Res. Cell Motil. 40 (2), 127–140. 10.1007/s10974-019-09520-2 31165949 PMC6726668

[B20] Hepple R. T. (1997). A new measurement of tissue capillarity: the capillary-to-fibre perimeter Exchange index. Can. J. Appl. Physiol. 22 (1), 11–22. 10.1139/h97-002 9018404

[B21] Hester G. M. VanDusseldorp T. A. Ha P. L. Kiani K. Olmos A. A. Jabbari M. (2022). Microbiopsy sampling for examining age-related differences in skeletal muscle fiber morphology and composition. Front. Physiology 12, 756626. 10.3389/fphys.2021.756626 PMC8784837 35082686

[B22] Jennekens F. G. I. Tomlinson B. E. Walton J. N. (1971). Data on the distribution of fibre types in five human limb muscles. An autopsy study. J. Neurol. Sci. 14 (3), 245–257. 10.1016/0022-510x(71)90215-2 4109253

[B23] Johnson M. A. Polgar J. Weightman D. Appleton D. (1973). Data on the distribution of fibre types in thirty-six human muscles an autopsy study. J. Neurological Sci. 18 (1), 111–129. 10.1016/0022-510x(73)90023-3 4120482

[B24] Kadi F. Eriksson A. Holmner S. Butler-Browne G. S. Thornell L. E. (1999). Cellular adaptation of the trapezius muscle in strength-trained athletes. Histochem Cell Biol. 111 (3), 189–195. 10.1007/s004180050348 10094415

[B25] Kottlors M. Kirschner J. (2010). Elevated satellite cell number in duchenne muscular dystrophy. Cell Tissue Res. 340 (3), 541–548. 10.1007/s00441-010-0976-6 20467789

[B26] Kriketos A. D. Baur L. A. O’connor J. Carey D. King S. Caterson I. D. (1997). Muscle fibre type composition in infant and adult populations and relationships with obesity. Int. J. Obes. Relat. Metab. Disord. 21 (9), 796–801. 10.1038/sj.ijo.0800476 9376893

[B27] Lee W. S. Hoi Cheung W. Qin L. Tang N. Leung K. S. (2006). Age-associated decrease of type IIA/B human skeletal muscle fibers. Clin. Orthop. Relat. Res. 450, 231–237. 10.1097/01.blo.0000218757.97063.21 16691139

[B28] Lexell J. Sjöström M. Nordlund A.-S. S. Taylor C. C. (1992). Growth and development of human muscle: a quantitative morphological study of whole vastus lateralis from childhood to adult age. Muscle and Nerve 15 (3), 404–409. 10.1002/mus.880150323 1557091

[B29] Lipina C. Hundal H. S. (2017). Lipid modulation of skeletal muscle mass and function. J. Cachexia, Sarcopenia Muscle 8, 190–201. 10.1002/jcsm.12144 27897400 PMC5377414

[B30] Mackey A. L. Andersen L. L. Frandsen U. Suetta C. Sjøgaard G. (2010). Distribution of myogenic progenitor cells and myonuclei is altered in women with vs. Those without chronically painful trapezius muscle, J. Appl. Physiol. 109 (6), 1920–1929. 10.1152/japplphysiol.00789.2010 20930124

[B31] Mackey A. L. Kjaer M. Charifi N. Henriksson J. Bojsen-Moller J. Holm L. (2009). Assessment of satellite cell number and activity status in human skeletal muscle biopsies. Muscle Nerve 40 (3), 455–465. 10.1002/mus.21369 19705426

[B32] Maier F. Bornemann A. (1999). Comparison of the muscle fiber diameter and satellite cell frequency in human muscle biopsies. Muscle Nerve 22 (5), 578–583. 10.1002/(sici)1097-4598(199905)22:5<578::aid-mus5>3.0.co;2-t 10331356

[B33] McGuire B. J. Secomb T. W. (2003). Estimation of capillary density in human skeletal muscle based on maximal oxygen consumption rates. Am. J. Physiol. Heart Circ. Physiol. 285 (6), 2382–2391. 10.1152/ajpheart.00559.2003 12893642

[B34] Oertel G. (1988). Morphometric analysis of normal skeletal muscles in infancy, childhood and adolescence. An autopsy study. J. Neurological Sci. 88 (1–3), 303–313. 10.1016/0022-510x(88)90227-4 3225628

[B35] Pallafacchina G. Blaauw B. Schiaffino S. (2013). “Role of satellite cells in muscle growth and maintenance of muscle mass,” in Nutrition, metabolism and cardiovascular diseases (Amsterdam, Netherlands: Elsevier). 10.1016/j.numecd.2012.02.002 22621743

[B36] Patel S. P. Gamboa J. L. McMullen C. A. Rabchevsky A. Andrade F. H. (2009). Lower respiratory capacity in extraocular muscle mitochondria: evidence for intrinsic differences in mitochondrial composition and function. Investigative Ophthalmol. Vis. Sci. 50 (1), 180–186. 10.1167/iovs.08-1911 PMC2615070 18791171

[B37] Pontén E. M. Stål P. S. (2007). Decreased capillarization and a shift to fast myosin heavy chain IIx in the biceps brachii muscle from young adults with spastic paresis. J. Neurological Sci. 253 (1–2), 25–33. 10.1016/j.jns.2006.11.006 17196619

[B38] Qaisar R. Bhaskaran S. Van Remmen H. (2016). Muscle fiber type diversification during exercise and regeneration. Free Radic. Biol. Med. 98, 56–67. 10.1016/j.freeradbiomed.2016.03.025 27032709

[B39] Rose J. Haskell W. L. Gamble J. G. Hamilton R. L. Brown D. A. Rinsky L. (1994). Muscle pathology and clinical measures of disability in children with cerebral palsy. J. Orthop. Res. Official Publ. Orthop. Res. Soc. 12 (6), 758–768. 10.1002/jor.1100120603 7983551

[B40] Saito Y. (1985). Muscle fibre type differentiation and satellite cell population in werdnig-hoffmann disease. J. Neurological Sci. 68 (1), 75–87. 10.1016/0022-510x(85)90051-6 3989581

[B41] Sallum A. M. E. Varsani H. Holton J. L. Marie S. K. N. Wedderburn L. R. (2013). Morphometric analyses of normal pediatric brachial biceps and quadriceps muscle tissue. Histol. Histopathol. 28 (4), 525–530. 10.14670/HH-28.525 23392619 PMC3740497

[B42] Simoneau J.-aime Claude B. (1989). Human variation in skeletal muscle fiber-type proportion and enzyme activities. Am. J. Physiol. 257 (4 Pt 1), E567–E572. 10.1152/ajpendo.1989.257.4.E567 2529775

[B43] Staron R. S. Hagerman F. C. Hikida R. S. Murray T. F. Hostler D. P. Crill M. T. (2000). Fiber type composition of the vastus lateralis muscle of young men and women. J. Histochem. Cytochem. 48 (5), 623–629. 10.1177/002215540004800506 10769046

[B44] Valentine J. Dyke J. Ward R. Thornton A. Blair E. Stannage K. (2019). Normative data of muscle fiber diameter of vastus lateralis during childhood: a field test. Muscle Nerve 59, 590–593. 10.1002/mus.26426 30680744

[B45] Verdijk L. B. Snijders T. Drost M. Delhaas T. Kadi F. Loon L. J. C. V. (2014). Satellite cells in human skeletal muscle; from birth to old age. Age 36 (2), 545–547. 10.1007/s11357-013-9583-2 24122288 PMC4039250

[B46] Wüst R. C. I. Gibbings S. L. Degens H. (2009). Fiber capillary supply related to fiber size and oxidative capacity in human and rat skeletal muscle. Oxyg. Transp. Tissue XXX 645, 75–80. 10.1007/978-0-387-85998-9_12 19227453

[B47] Zeller-Plumhoff B. Roose T. Clough G. F. Schneider P. (2017). Image-based modelling of skeletal muscle oxygenation. J. R. Soc. 14 (127), 20160992. 10.1098/rsif.2016.0992 PMC5332585 28202595

[B48] Zoladz J. A. Semik D. Zawadowska B. Majerczak J. Karasinski J. Kolodziejski L. (2005). Capillary density and capillary-to-fibre ratio in vastus lateralis muscle of untrained and trained men. Folia Histochem. Cytobiol. 43 (1), 11–17. 15871557

