# Correction: Histological analysis of the medial gastrocnemius muscle in young healthy children

**DOI:** 10.3389/fphys.2025.1653150

**Published:** 2025-07-16

**Authors:** Anke Andries, Jorieke Deschrevel, Karen Maes, Nathalie De Beukelaer, Marlies Corvelyn, Lauraine Staut, Hannah De houwer, Domiziana Costamagna, Stefaan Nijs, Willem-Jan Metsemakers, Elga Nijs, Greet Hens, Eva De Wachter, Sandra Prinsen, Kaat Desloovere, Anja Van Campenhout, Ghislaine Gayan-Ramirez

**Affiliations:** ^1^ Laboratory of Respiratory Diseases and Thoracic Surgery, Department of Chronic Diseases and Metabolism, KU-Leuven, Leuven, Belgium; ^2^ Neurorehabilitation Group, Department of Rehabilitation Sciences, KU-Leuven, Leuven, Belgium; ^3^ Stem Cell and Developmental Biology, Department of Development and Regeneration, KU-Leuven, Leuven, Belgium; ^4^ Pediatric Orthopedics, Department of Development and Regeneration, KU-Leuven, Leuven, Belgium; ^5^ Exercise Physiology Research Group, Department of Movement Sciences, KU-Leuven, Leuven, Belgium; ^6^ Department of Trauma Surgery, University Hospitals Leuven, Leuven, Belgium; ^7^ Department of Ear Nose Throat, University Hospitals Leuven, Leuven, Belgium; ^8^ Department of Orthopaedic Surgery, University Hospitals Leuven, Leuven, Belgium

**Keywords:** boys, girls, muscle capillaries, muscle fiber size and proportion, myosin heavy chain, satellite cells, fiber size variability

In the published article, there was an error in the **Funding statement**. The funder research Foundation-Flanders, FWO grant G084523N to the published study was erroneously omitted.

The original text was: “The author(s) declare financial support was received for the research, authorship, and/or publication of this article. This study was funded by an internal KU Leuven grant (C24/18/103) and by Research Foundation–Flanders (FWO grant G0B4619N)”. The correct Funding statement appears below.

